# SH-DETR: Enhancing steel surface defect detection and classification with an improved transformer architecture

**DOI:** 10.1371/journal.pone.0334048

**Published:** 2025-11-11

**Authors:** Shouluan Wu, Hui Yang, Liefa Liao, Chao Song, Yating Fang, Yang Yang

**Affiliations:** 1 Jiangxi University of Science and Technology, Nanchang, Jiangxi, China; 2 Jiangxi Modern Polytechnic College, Nanchang, Jiangxi, China; 3 HeBei University of Architecture, Zhangjiakou, Hebei, China; Jiangsu Open University, CHINA

## Abstract

The detection of defects on steel surfaces constitutes a vital area of research in computer vision, characterized by its complexity and variety, which pose significant difficulties for accurate identification. In this context, we introduce a deep learning framework that combines multi-channel random coding with modules for multi-scale feature fusion to tackle the challenges of low recognition accuracy and insufficient classification power prevalent in conventional models. Our model capitalizes on the self-attention mechanism associated with the Transformer architecture, alongside the strong feature extraction capabilities of Convolutional Neural Networks (CNNs), to facilitate a combined improvement in performance. To start, we enhance the model’s feature extraction functionality by incorporating ResNet18 along with global self-attention. Next, we bring forth a novel improvement to the backbone network by adding a multi-channel shuffled encoding module, which effectively encodes various features through the interactions of different convolutional groups, thus minimizing the number of parameters. Additionally, we introduce a multi-feature fusion module UPC-SimAM (upsample concatenated Simple Parameter-Free Attention Module), which is free from parameter redundancy to bolster the model’s capacity to merge multi-scale features. Our experiments on the NEU-DET and GC10-DE datasets demonstrate that our model outperforms existing state-of-the-art techniques regarding detection efficiency. Specifically, the model registers a classification accuracy of 91.72%, an mAP@0.5 of 83.03%, and an mAP@0.5:0.95 of 45.55% on the NEU-DET dataset. On the GC10-DE dataset, it achieves a classification precision of 76.73%, an mAP@0.5 of 65.03%, and an mAP@0.5:0.95 of 32.46%. Through detailed ablation studies and visualization experiments, we affirm the considerable potential and benefits of the proposed SH-DETR model in the field of detecting defects on steel surfaces.

## Introduction

Steel is a fundamental raw material in industrial production and plays an indispensable role. However, during practical manufacturing, steel surfaces may develop defects such as corrosion or deformation, making the detection of these surface defects critical [1]. Common defect types include scratches and inclusions, which can severely impact the performance of the steel. The quality of steel is closely related to these surface defects, directly affecting the safety and reliability of the final products [2,3]. With societal development and increasing demands for higher industrial material quality, the quality of materials has become a focal point of attention [4]. Traditional manual detection approaches face several constraints, especially in extensive production contexts, where their effectiveness and dependability frequently do not meet current industrial needs [5]. Although automatic detection methods using advanced algorithms have improved performance, early techniques such as threshold segmentation remain sensitive to environmental factors and cannot achieve the desired accuracy. Consequently, accurately identifying surface defects on steel and realizing efficient steel inspection have emerged as urgent issues that need to be addressed within the industry [6].

The swift progress of deep learning technology has resulted in the extensive implementation of object detection methods based on deep learning across a variety of fields, due to their benefits in terms of stability, safety, efficiency, real-time capabilities, and precision. These techniques are deemed essential solutions for businesses seeking to attain automation and intelligent operations [7]. Neural networks are frequently utilized to identify performance flaws in steel structures. Nevertheless, augmenting the number of layers within the network may result in issues such as vanishing gradients and overfitting. To tackle these challenges, Targ *et al*. introduced the ResNet architecture, which successfully alleviates the degradation issue often encountered during the training processes of deep networks [8]. Presently, object detection algorithms that leverage deep learning are mainly divided into two categories: two-stage and one-stage object detectors. Researchers have proposed a novel two-stage framework named PSSCL. It combines GCE loss with contrastive learning. This design effectively reduces the overfitting problem caused by noisy labels. PSSCL also achieves significant performance improvements on various noisy datasets [9]. Examples of two-stage object detectors encompass R-CNN (Region-based Convolutional Neural Network), Fast R-CNN, and Faster R-CNN [10,11]. On the other hand, one-stage object detectors predominantly include SSDs (Single-Shot MultiBox Detectors) and YOLO (You Only Look Once) [12]. Although two-stage object detectors are recognized for their high accuracy, they generally operate at a slower pace compared to their one-stage equivalents. The SSD algorithm conducts object detection through multiple convolutional layers and feature maps, where each feature map is linked to a particular set of predefined default boxes. While it maintains a balance between efficiency and accuracy, it encounters difficulties with the detection of intricate objects. Conversely, the YOLO algorithm redefines the object detection task as a regression issue by segmenting the complete input image into a grid, which enables simultaneous processing of the entire image instead of performing numerous detections at different scales or locations [13]. Researchers introduced a spatial attention mechanism without dimensionality reduction. This mechanism helps precisely locate key regions. They also applied depthwise separable convolutions to reduce the number of parameters. In addition, Dropout regularization and data augmentation were used. Together, these strategies provide an efficient and low-complexity object recognition solution [14]. Zhang *et al*. [15] proposed a noise-label learning framework. It combines a balanced partition mechanism with a pseudo-label relaxed contrastive loss. The balanced partition addresses class imbalance in sample distribution. Oversampling and contrastive loss optimization further reduce conflicts in semi-supervised learning. As a result, the framework achieves robust performance that is optimal or nearly optima. This methodology offers a distinct benefit regarding speed and accuracy, and the suite of algorithms continues to evolve and enhance [16].

Recognized for its outstanding capability in managing sequential data, the Transformer has found widespread use across diverse fields, including computer vision. In industrial object detection, the Transformer’s encoder and self-attention mechanisms are especially valued for their strong feature synthesis capabilities. This has led to the emergence of feature aggregation networks as a new paradigm for object detection [17]. The Swin Transformer enhances global attention modeling through a windowed self-attention strategy. This approach reduces the computational complexity from a quadratic to a linear relationship with image size, thereby achieving state-of-the-art performance in downstream tasks. The Swim Transformer improves the model’s global attention capabilities by employing a windowed self-attention strategy, which shifts computational complexity from a quadratic correlation with image dimensions to a linear one, thus attaining state-of-the-art performance in subsequent tasks. In the arena of steel surface defect detection, defects vary in shape and size, including elongated, irregular, and expansive forms. Conventional CNNs frequently face challenges in capturing long-range dependencies in visual tasks, while the Transformer’s capacity to handle such dependencies provides a significant edge in overcoming this issue [18,19]. The positional encoding within the encoder aids in the localization and extraction of essential features from the feature map, thus enhancing the retrieval of feature information [20]. Nevertheless, simply applying the Transformer to steel defect detection tasks is inadequate and necessitates modifications tailored to its distinctive traits. As a result, this research introduces specific enhancements to the Transformer model to ensure it meets the unique demands of identifying defects on steel surfaces more effectively.

## Related work

### CNN method

Fueled by advancements in deep learning, the domain of industrial defect detection has experienced a surge of innovative methodologies. The newly introduced Multi-Level Feature Fusion Network (MFN) facilitates the incorporation of features from multiple layers, encompasses detailed semantic information, and emphasizes areas of interest via the Regional Suggestion Network (RPN), thereby propelling research forward in the area of steel defect detection [21]. Akhyar *et al*. adopted R-CNN as their foundational network architecture and enhanced model performance in defect localization by implementing various convolution enhancements and RoI pooling methods to effectively identify different defect types and sizes [22]. To further boost detection rates, Li *et al*. initially increased the dataset size and subsequently developed a multi-layer feature fusion network grounded in Faster R-CNN for the identification of surface defects [23].

### One-stage detection method

In contrast to methods utilizing two-stage detection, one-stage algorithms like SSD and YOLO directly predict the locations of bounding boxes and the categories of objects within the network framework [24]. This strategy eliminates the necessity for distinct candidate regions, thereby greatly enhancing the speed of the detection process. Building on the architecture of RetinaNet, Akhyar *et al*. integrated a feature pyramid network and refined the loss function, which effectively tackled the difficulties of recognizing targets with limited instances. To further improve the precision of defect classification and localization, Cheng *et al*. introduced an innovative channel attention mechanism derived from RetinaNet, which successfully reduced the loss of vital information. They also developed a spatial feature fusion module designed to combine features at various levels, encompassing both shallow and deep attributes [25]. After achieving improved accuracy, researchers began to prioritize enhancing speed while maintaining accuracy. Tian *et al*. presented a pioneering feature extension augmentation model that expands the receptive field of the model and employs a new central function to achieve more accurate localization and detection of target points [26]. Additionally, Kou *et al*. integrated several dense convolutions into the YOLO model, with experimental findings showing that this convolution method can substantially boost feature information extraction and enhance network performance [27].

### Improved approach based on transformer

Researchers are increasingly focused on enlarging the receptive field and extracting essential features, which has led to a shift in research emphasis towards learning intricate textures. Guo *et al*. incorporated Transformers into the backbone network and inspection heads of YOLO, allowing these heads to adapt dynamically to defects of multiple sizes [28]. Meanwhile, Yang *et al*. utilized Real-ESRGAN technology to boost image resolution, successfully tackling the challenge of recognizing small defects in steel datasets. They also developed an attention module rooted in CBAM and SCSE, which greatly enhanced the interaction and fusion of both channel and spatial information [29]. In another study, Zhao *et al*. introduced the RT-DETR model to address the lack of interaction and fusion among multi-scale features. The model promotes feature integration across different scales, which accelerates both training and detection while also improving accuracy by reducing uncertainty in query selection [30,31]. Lv *et al*. proposed the MobileViT v2-YOLO v8 network, which enhances the model’s capability to extract features from complex defect shapes by integrating deep and shallow features through additional convolutional layers. This approach effectively combines the strengths of Convolutional Neural Networks (CNNs) and Transformers [32]. In a related study, Mao *et al*. substituted the BasicBlock module in RT-DETR with a lightweight MobileNetV3 module, enabling comprehensive capture of both long-range and local feature interactions of steel defects. Furthermore, they incorporated more efficient depthwise separable convolution (DWConv) and VoVGSCSP structures into RT-DETR, refining the feature fusion network. These modifications resulted in improved feature extraction and fusion, reduced computational complexity, and a lightweight architecture [33]. Collectively, these advancements in research provide valuable insights and innovative techniques for the domain of industrial defect detection.

### Our contributions

We address the limitations of existing models regarding accuracy and specific location information by proposing a novel Transformer-based multi-feature fusion network for steel surface defect detection, named SH-DETR. Our primary contributions are as follows:

1. We integrate an encoder, decoder, global self-attention mechanism, and Convolutional Neural Network (ResNet18), enabling more precise defect recognition;
2. We designed a novel SH-encoder module that not only reduces model parameters but also effectively addresses the lack of interaction between different convolutional groups, thereby improving the efficiency and effectiveness of feature fusion;
3. We introduced an efficient feature extraction module (UPC-SimAM), which facilitates energy-weighted fusion of multi-scale features across branches with varying kernel sizes without increasing the number of parameters, further enhancing the model’s capacity to capture intricate details.

These contributions not only advance the technology of steel surface defect detection but also offer new ideas and methods for applying deep learning in industrial vision inspection. Through these innovations, the SH-DETR model demonstrates significant advantages in both detection accuracy and computational efficiency.

## Methods

### SH-encoder

**Fig 1:**
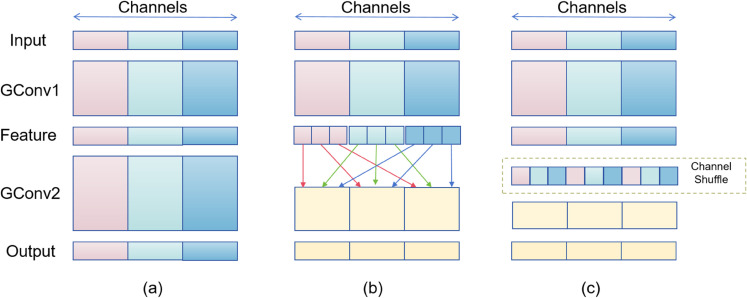
Channel shuffle flowchart. (a) shows the original grouped convolution, (b) shows different channels being shuffled to different positions after grouped convolution, and (c) shows the result after channel shuffling is completed.

In this study, we propose the SH-encoder, which utilizes channel shuffling followed by a layer of Transformer-based encoding transformation and a self-attention mechanism, as illustrated in Fig 1. This design is specifically tailored to process the final CNN feature output produced by the backbone network. Traditional convolutional operations are typically restricted to their designated channel groups, which limits the exchange of information across different channels. By implementing channel shuffling, we rearrange the channel order, thereby promoting information exchange between various channel groups and enhancing the feature extraction capabilities. Figure (a) illustrates the conventional convolution operation. It can be observed that without additional operations, the output features are computed based on only a subset of the input channels. This operation hinders the flow of information, thereby reducing the expressive power of the features. Therefore, we hope to fuse the channel information between feature maps after group convolution, as shown in Figure (b). By distributing the features of each group to different groups and then performing convolution, the output features can incorporate the characteristics of all groups, as demonstrated in Figure (c).

Channel shuffle can cleverly achieve this operation through conventional tensor operations. To facilitate inter-group information exchange, the input feature map with dimensions C×H×W (where *C* denotes the number of channels) is first reshaped into a four-dimensional tensor according to the number of groups *g* and the number of channels per group *n*, such that C=g×n. The reshaping operation is expressed as:

$$X_{\text{reshaped}}=\text{reshape}(X_{g,n,H,W}),\;X\in {\mathbb{R}}^{g\;\times \;n\;\times \;H\;\times \;W}$$ (1)

To simplify the spatial dimensions, H×W is flattened into a single dimension *s*, reducing the tensor to: $X\in {\mathbb{R}}^{g\;\times \;n\;\times \;s}$. Next, a transposition is performed across the group and channel axes to shuffle the feature channels, enabling cross-group communication. The transposed tensor is defined as:

$$X_{\text{transposed}}=X_{\text{reshaped}}^{T},\;X\in {\mathbb{R}}^{g\times n\times s}$$ (2)

Following the transposition, the tensor is reshaped back to the original three-dimensional format by merging the *n* and *g* dimensions:

$$X_{\text{shuffled}}=\text{reshape}(X_{\text{transposed}}[g,n,H,W]),\;X_{\text{shuffled}}\in {\mathbb{R}}^{C\times H\times W}$$ (3)

At this stage, the channel order has been shuffled, effectively mixing the information across different groups. Finally, a group-wise convolution is applied to the shuffled feature map, where each group independently performs a 1×1 convolution to further integrate the features and enhance inter-channel interaction.

The model can effectively learn and depict defect characteristics due to this innovation, which also decreases the number of parameters as well as the computational effort involved [34]. We flatten the features of the feature map into high-dimensional vectors, which are then passed to the encoder for processing, transforming multi-scale features into sequences (RB × C × H × W → RB × N × C) and concatenating them into vectors of sequence length. Subsequently, we leverage self-attention technology to facilitate multi-scale feature interaction. The self-attention mechanism emphasizes the semantic nature of features; compared to the shallower features in CNN networks, deeper features contain richer and more advanced semantic information. Consequently, the Encoder processes only high-level features, which not only significantly reduces computational load and enhances processing speed but also maintains performance.

**Fig 2:**
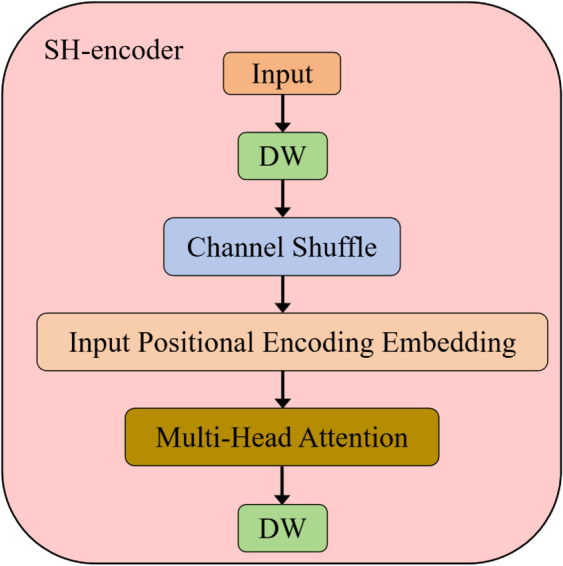
SH-encoder module, DW stands for depthwise separable convolution.

In the process of flattening the S5 feature map, the input embedding vector is transformed into three distinct vectors: Query, Key, and Value, as illustrated in Equation 4. For each input, the Query vector is computed by taking the dot product with all Key vectors, resulting in attention weights. This mechanism enables each element in the input sequence to focus on other elements at varying positions, thereby effectively capturing internal dependencies within the sequence. The attention weights are subsequently normalized using the Softmax function and multiplied by the corresponding Value vectors to generate the output vector. Given that the encoder does not incorporate convolutional or recurrent structures to inherently capture the order of the sequence, it is crucial to include explicit positional information. To address this, positional encoding is utilized to append the positional information of each element in the input sequence to its corresponding embedding vector. We append a learned positional embedding to the sequence at each position. The self-attention mechanism (SA(Q, K, V)) is illustrated in Equation 5. The multi-head attention mechanism captures various dependencies by utilizing multiple independent heads. The outputs of these heads are subsequently merged and processed through a linear layer, ultimately generating the output of the attention mechanism. Finally, we reshape the output back to two dimensions, as indicated in Equation 6, to facilitate subsequent cross-scale feature fusion. This mechanism enables the self-attention within the encoder to capture global dependencies within the sequence without relying on the order of the sequence, thereby enhancing the model’s robustness and generalization capabilities. Fig 2 illustrates the structure of the SH-encoder

$$Q=K=V=Flatten(S_{5})$$ (4)

$${SA}^{l}(Q,K,V)=E_{in}^{l}+softmax\left(\frac{Q^{l}x{(K^{l})}^{T}}{\sqrt{d}}\right)V^{l}$$ (5)

$$F_{5}=Reshape\left(Attn\left(Q,K,V\right)\right)$$ (6)

### UPC-SimAM

**Fig 3:**
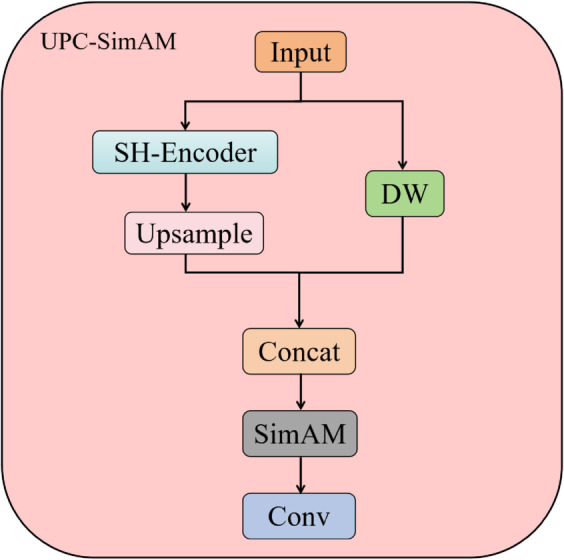
UPC-SimAM module, DW stands for depthwise separable convolution.

This research focuses on the intricacies of defects on steel surfaces and the concentration of minor target imperfections by introducing UPC-SimAM aimed at enhancing feature processing in the backbone network. While deep networks are capable of extracting rich global features, the redundancy inherent in deep channel information often leads to an increase in model size and a decrease in the model’s focus on densely packed small targets. Furthermore, traditional feature fusion methods, such as simple addition or concatenation, frequently yield unsatisfactory results. To address this, UPC-SimAM employs a weighted fusion strategy that enhances the model’s attention to target regions, thereby improving feature fusion efficiency and reducing the redundancy of noisy features. In existing models, it is common practice to first adjust the scale of feature maps through upsampling, followed by the fusion of features from different branches or scales via concatenation. This approach often results in a significant amount of redundant features, which may obscure some less prominent yet important features. In contrast, UPC-SimAM adopts a weighted fusion mechanism to integrate feature maps from various scales. We applied two parallel strategies to the input feature maps. First, channel shuffling was used to promote interaction and fusion between different feature maps. At the same time, depthwise separable convolution was applied to reduce computational parameters while preserving effective features. Subsequently, we concatenated the feature maps processed by the aforementioned operations. Building upon this, we introduced our SimAM attention mechanism, which highlights key features. At each fusion node, multiple input feature maps are combined through a weighted summation, with the weighting coefficients being automatically learned by the network and treated as learnable parameters, as illustrated in Fig 3. These parameters regulate how features contribute at various scales, making certain that the more significant or distinguishing features are assigned higher weight in the fusion process, which in turn allows for a more efficient capture of multi-scale information regarding targets. During the convolution operation, the highlighted features can be integrated, further enhancing the model’s expressiveness. This module enables the model to maintain low complexity while achieving excellent performance. The results of the ablation experiments presented below underscore the unique advantages of the proposed module compared to other contemporary methods, particularly in its ability to avoid excessive parameter overhead.

**Fig 4:**
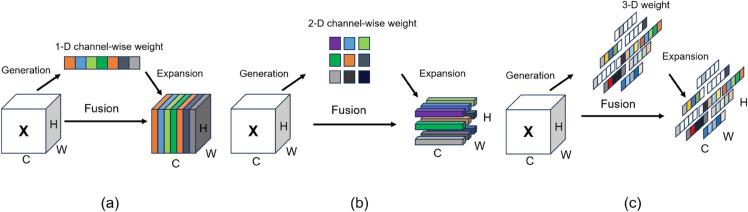
SimAM attention. (a) Channel-wise attention, (b) Spatial-wise attention, (c) Full 3-D weights for attention.

SimAM creates attention weights by evaluating the similarity of each pixel in the feature map with its neighboring pixels, operating under the premise that pixels close to one another in an image demonstrate a high degree of similarity, whereas pixels that are farther apart display lower similarity. Unlike existing channel and spatial attention modules [35], SimAM serves as a lightweight attention mechanism that enhances the performance of convolutional neural networks (CNNs) through a simplified approach. This module does not require additional parameters and imposes minimal computational burden, yet it effectively captures key feature information, as illustrated in Fig 4. It improves CNN performance by calculating local self-similarity within the feature map. The calculation of feature image pixels is shown in Equation 7. To extract the feature map from the input image using a CNN, where is the batch size, is the number of channels, and and are the height and width of the feature map, respectively. For each pixel in the feature map (where and are the position indices of the pixel in the feature map), SimAM calculates its similarity with the surrounding pixels. This similarity is measured by the distance between the feature vectors of the pixels, with the negative square of the Euclidean distance being a common choice. However, SimAM actually reflects the similarity indirectly by calculating the average of the squared differences between each pixel and its neighboring pixels (after normalization). Specifically, for each pixel, the squared differences with all the pixels in its neighborhood are calculated, then summed and normalized.

$$S_{i,j}=\frac{1}{N}\sum_{k\in \Omega_{i,j}}\|{𝐱}_{i,j}-{𝐱}_{k}{\|}_{2}^{2}$$ (7)

$$\omega_{i,j}=1/\left(1+\exp\left(-\frac{1}{4}\left(\frac{s_{i,j}}{\sigma_{i,j}^{2}}-1\right)\right)\right)$$ (8)

Here $\omega_{i,j}$ represents the neighborhood of pixel $x_{i,j}$ (excluding $x_{i,j}$ itself), and N is the number of pixels in the neighborhood. However, in the actual implementation of SimAM, the entire feature map’s mean is typically used for centralization, and the squared difference is calculated by subtracting the centralized result to simplify the computation. Based on the calculated *S*_*i*,*j*_ (more precisely, based on the squared difference after centralization), SimAM generates the attention weight $\omega_{i,j}$ using the Equation 8.

In Equation 8, $\sigma_{i,j}^{2}$ is a form of normalization for *S*_*i*,*j*_ (in the implementation of SimAM, it is usually approximated by the average and standard deviation of *S*_*i*,*j*_ over the entire feature map or a local region), and ∈ is a very small constant (such as 1×10-4) to prevent division by zero. This formula is actually a variant of the sigmoid function, used to map *S*_*i*,*j*_ to the interval (0, 1) as the attention weight. The generated attention weight map $W\in {\mathbb{R}}^{B\;\times \;1\;\times \;H\;\times \;W}$ (here the channel dimension is ignored because SimAM typically calculates the attention weight independently for each channel) is multiplied with the original feature map *X* to obtain the weighted feature map $X^{\prime }=W⊙X$, where ⊙ denotes element-wise multiplication. The module’s operation primarily depends on the chosen energy function, thereby avoiding excessive structural modifications and eliminating the need for additional parameters to derive 3D attention weights for feature graphs. It employs binary labels in conjunction with regularization terms, utilizing the energy of each pixel in the feature map to assess its contribution to the model’s task. Specifically, the minimum energy can be computed from Equation 9.

In Equation 9, *λ* serves as the regularization term to constrain the size of certain parameters in the model, while *t*_*k*_ corresponds to the k-th neuron within a single channel of the input feature map. The symbol $\hat{u}$ denotes the mean of all neurons across a single channel, and ${\hat{\sigma }}^{2}$ signifies the variance of all neurons within the same channel. A lower $e_{t}^{*}$ value indicates reduced energy.The more distinct neuron k is from its adjacent neurons, the higher its importance, and the greater its significance in visual processing tasks.

The computation of the output feature map $\tilde{X}$ is detailed in Equation 10. In this context, *E* encompasses the collection of all neurons, represents the input feature map, and sigmoid functions as an activation function. The purpose of this function is to limit the values of *E* and to perform feature enhancement. This mechanism enables UPC-SimAM to capture global dependencies within sequences irrespective of their order, thus bolstering the model’s robustness and generalization capabilities.

$$e_{t}^{*}=\frac{4(\sigma^{2}+\lambda )}{4(t_{k}-\hat{u}{)}^{2}+2\lambda +\sigma^{2}}$$ (9)

$$\tilde{𝐗}=sigmoid\left(\frac{1}{E}\right)\cdot 𝐗$$ (10)

### SH-DETR

**Fig 5:**
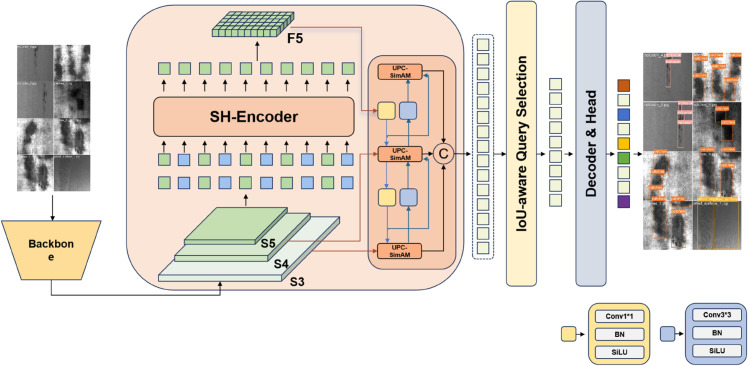
SH-DETR architecture.

In this research, we introduce SH-DETR, a robust and effective single-stage framework for object detection that merges the advantages of real-time operation, precision, and stability. Unlike previous models, SH-DETR enables real-time, end-to-end object detection without the need for any post-processing, thus maintaining consistent speed during inference and preventing additional lags. Moreover, the model utilizes a query selection algorithm based on Intersection over Union (IoU), which notably improves performance and provides a more effective method for initializing the target query. The foundational architecture of SH-DETR consists of four key elements: a backbone network, a hybrid encoder, multi-feature fusion, and a converter decoder with an auxiliary prediction head. Together, these elements form the structure of the SH-DETR framework, guaranteeing its effective performance and accurate capabilities in object detection. The specific architecture of SH-DETR is illustrated in Fig 5.

The primary function of the backbone network is feature extraction, which predominantly consists of the Convolution-Batch Normalization (ConvBN) module and the Basic Block module. The ConvBN module integrates the convolutional layer with the batch normalization layer, effectively expanding the network’s receptive field. Meanwhile, the Basic Block module, based on the ResNet architecture, is structured with two convolutional layers and residual connections. This design not only mitigates the issue of vanishing gradients but also improves the model’s expressiveness and overall performance.

Our model introduces an SH-encoder module designed to address the high computational burden and gradient descent challenges associated with traditional convolution methods. We propose a technique that enhances the capture and retention of local feature details by integrating grouped convolution with channel shuffling. This approach not only improves the model’s accuracy and robustness but also reduces its complexity and the number of parameters. Decoders typically exhibit high computational complexity and low efficiency. To mitigate this issue, we employ a multi-head segmentation module that divides the feature map into multiple segments, subsequently inputting the shuffled feature vectors into the encoder and the self-attention module to establish correlations and embed positional information. This strategy enhances global attention while reducing the original computational burden to a linear level. Furthermore, we introduce a channel shuffling module to address the issues of fixed positions and limited interconnections within the image encoding channel, significantly improving the efficiency and accuracy of the visual encoder when handling complex visual tasks. As a feature fusion component of the network, the AIFI encoder incorporates an Internal Scale Feature Interaction (AIFI) module based on cross-self-attention. This module extracts rich high-level semantic information through a single-layer encoder and effectively captures the relationships between conceptual entities within the image.

The cross-scale feature fusion module of UPC-SimAM effectively leverages features from diverse levels for integration. This fusion module includes two 1×1 convolutional layers alongside several RepBlock components, making full use of the combined advantages of features across varying scales. By integrating the encoded feature vectors into the internal scale feature interaction module, the network significantly enhances its ability to capture both global dependencies and complex local details within images. During the upsampling process, the cross-scale fusion module combines feature maps produced by other modules; these feature maps maintain particular positional information, which aids in more efficient feature extraction and multi-frequency fusion. Furthermore, the incorporation of the UPC-SimAM mechanism allows for dynamic adjustment of the focus on important features, enabling accurate localization of defect regions and notably enhancing the efficiency and precision of subsequent decoding tasks in complex visual applications. The multi-feature fusion approach consolidates initial feature maps from shallow to deep layers, increasing attention levels across the feature maps at each stage while maintaining fine details, thereby improving the detection of subtle characteristics.

Utilizing an IoU-aware query mechanism, a defined quantity of image features is chosen from the output sequence resulting from feature fusion to function as the initial target queries for the decoder. The decoder incorporates supplementary prediction heads that consistently refine these target queries, producing accurate bounding boxes and confidence scores. This approach successfully tracks defects, thus boosting the detector’s overall performance. As a result, this effective model markedly increases the detection accuracy for minor defects.

### Loss function

L1 loss, also known as Mean Absolute Error (MAE), is widely used in regression tasks. This loss function measures the error by calculating the sum of the absolute differences between the model’s predicted values and the actual values(as shown in Equation 11). Compared to L2 loss (Mean Squared Error, MSE), L1 loss exhibits greater robustness to outliers because it is less sensitive to extreme values in the data. This characteristic makes L1 loss often provide more robust performance when dealing with datasets that contain outliers.

$$L_{1Loss}=\frac{1}{n}\sum_{i=1}^{n}\left|y_{i}-{\hat{y}}_{i}\right|$$ (11)

The *L*1 loss function measures the absolute difference, rather than the squared difference, between predicted values and actual values, showcasing a lower sensitivity to outliers. This feature makes *L*1 loss more resilient when extreme values are present in the dataset. Moreover, the gradient of *L*1 loss maintains a constant value of ±1, leading to infrequent updates in certain optimization methods and resulting in sparse adjustments. Nevertheless, *L*1 loss effectively indicates the average size of prediction errors and keeps the same units as the original data, thus enhancing interpretability. In areas such as image denoising, *L*1 loss contributes to maintaining the accuracy of pixel values in images, effectively preventing excessive penalties for significant errors.

GIoU loss (Generalized Intersection over Union Loss) is an advanced loss function employed in object detection tasks. Compared to the standard IoU loss, as shown in Equation 12, GIoU loss demonstrates enhanced performance in optimizing the accuracy of bounding box localization, particularly in situations where there is no overlap between bounding boxes, as it continues to provide a meaningful optimization signal. The application of this loss function in object detection models significantly enhances the model’s performance in bounding box regression. In this context, C represents the calculation of the area of the smallest enclosing rectangle that encompasses both the predicted box and the true box, referred to as the closure box area.

The GIoU metric ranges from [−1, 1], as illustrated in Equation 13. GIoU achieves a value of 1 when two bounding boxes are perfectly coincident and falls below 0 when the bounding boxes do not overlap at all. Consequently, the GIoU Loss, which ranges from [0, 2], indicates a higher degree of alignment between the predicted and true bounding boxes with lower values. Unlike the standard IoU Loss, which fails to generate effective gradients when bounding boxes do not intersect, thereby limiting the model’s learning capacity, GIoU Loss provides an optimization signal even in non-intersecting scenarios, thereby promoting faster model convergence. Compared to IoU Loss, GIoU Loss offers more stable gradient updates, facilitating more accurate regression to the target location. By reducing localization errors, GIoU Loss significantly enhances the model’s accuracy in predicting bounding boxes, as demonstrated in Equation 14.

$$IoU=\frac{\text{Area of Intersection}}{\text{Area of Union}}$$ (12)

$$GIoU=IoU-\frac{\text{Area of }C-\text{Area of Union}}{\text{Area of }C}$$ (13)

$$GIoU_{Loss}=1-GIoU$$ (14)

## Experiment

In this study, we carried out an extensive evaluation of SH-DETR’s efficiency using two different benchmark datasets. A thorough explanation of these datasets and the experimental conditions was provided to promote transparency and reproducibility in our evaluation methodology. To assess the proposed SH-DETR model’s effectiveness on the test datasets, we compared its performance to various cutting-edge techniques. To evaluate performance, we selected key metrics such as recognition precision, recall rate, mAP@0.5, and mAP@0.5:0.95, detailed in Equations 15-17, which effectively reflect the detection abilities of the model, in addition, we employ GFLOPs (Giga Floating Point Operations) and FPS (Frames Per Second) as evaluation metrics to assess the computational complexity of the model. The formulas for these calculations are described below. Moreover, we applied transfer learning to enhance the model further, leading to a substantial increase in the precision of the optimized version. These results not only validate the SH-DETR model’s effectiveness but also underscore its potential and versatility in object detection applications.

$$\text{Precision}=\frac{\text{TP}}{\text{TP}+\text{FP}}$$ (15)

$$\text{Recall}=\frac{\text{TP}}{\text{TP}+\text{FN}}$$ (16)

$$\text{mAP}=\frac{\sum_{i=1}^{N}\text{AP}}{N}$$ (17)

### Training equipment and testing procedure

In this section, we analyze the results of the SH-DETR model experiments. The model underwent training using the training dataset and was subsequently assessed with the test dataset to evaluate its effectiveness. When the dataset is not sufficiently large to permit a separation into distinct training, validation, and test datasets, we often apply an 80-20% data division approach, dedicating 80% of the data for training and the remaining 20% for validation. We will then present the results obtained from the NEU-DET and GC10-DET datasets. During the experiments conducted with these datasets, we employed the AdamW optimizer, with a learning rate set at 0.0001 and a momentum value of 0.9. To thoroughly evaluate the performance of the model, we relied on key metrics, such as recognition precision, recall rate, mAP@0.5, and mAP@0.5:0.95, to validate the model’s effectiveness. These metrics together provide insight into the model’s success in object detection tasks. In the experimental setup, the NEU-DET dataset was trained with an input size of 200×200, while the GC10-DET dataset used an input size of 640×640. Both datasets were trained for 250 epochs. The loss function combined GIoU loss with L1 loss, and no early stopping strategy was applied throughout the training process. All deep learning models were executed on a workstation equipped with an Intel(R) Xeon(R) Silver 4214R CPU running at 2.40 GHz with dual processors, 128 GB of RAM, and an NVIDIA GeForce RTX 3090 GPU. Experimental hardware and software configuration is shown in Table 1, which guaranteed the robustness and consistency of both model training and testing procedures.

**Table 1:** Experimental hardware and software configuration.

Experimental Environment	Configuration
GPU	NVIDIA GeForce RTX 3090
CPU	Intel(R) Xeon(R) Silver 4214R CPU, 2.40GHz
Deep Learning Framework	PyTorch 1.8.1
yy Python Version	Python 3.9
Epoch	500
Early Stopping Strategy	No

### Dataset details

**Fig 6:**
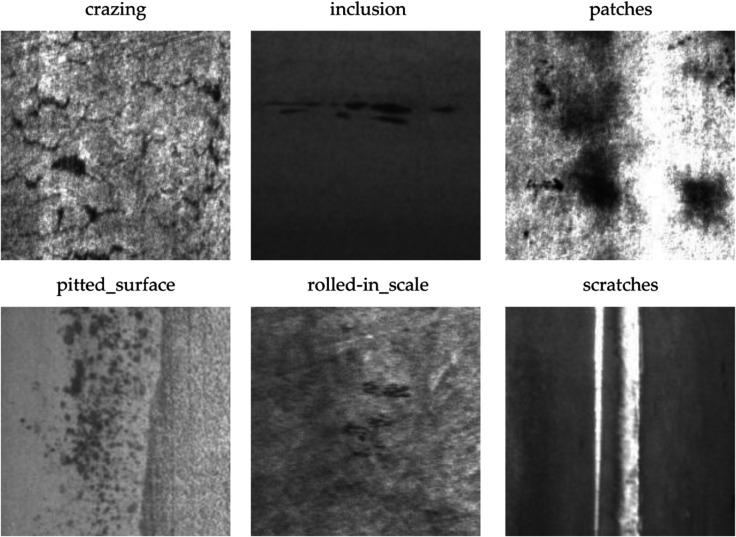
Examples of each category in NEU-DET.

**Fig 7:**
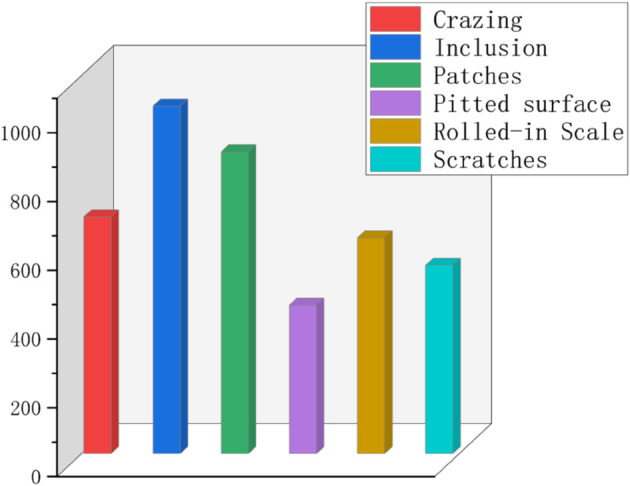
Distribution of categories in the NEU-DET dataset.

The NEU-DET [21] dataset was specifically developed for the purpose of identifying defects on steel surfaces. It is extensively utilized in machine vision and deep learning to develop and evaluate various algorithms associated with defect detection. In this research, the dataset comprises 1,800 images depicting various steel surface defects, all at a resolution of 200×200 pixels. It includes six unique categories of steel surface defects: Cracks, Patches, Inclusions, Pitted Surface, Crazing, and Scratches, as shown in Fig 6. The distribution of each defect category is illustrated in Fig 7.

**Fig 8:**
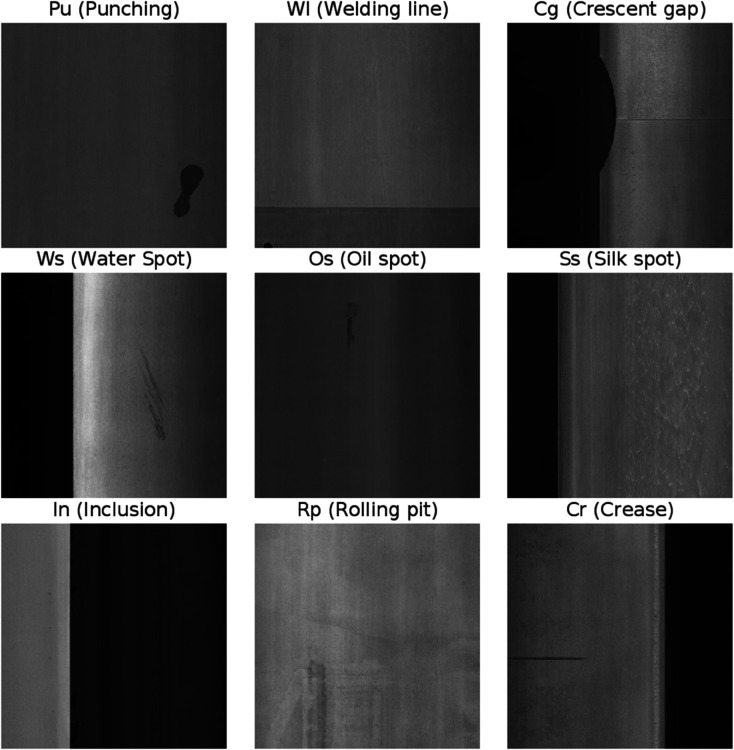
Examples of each category in GC10-DET.

**Fig 9:**
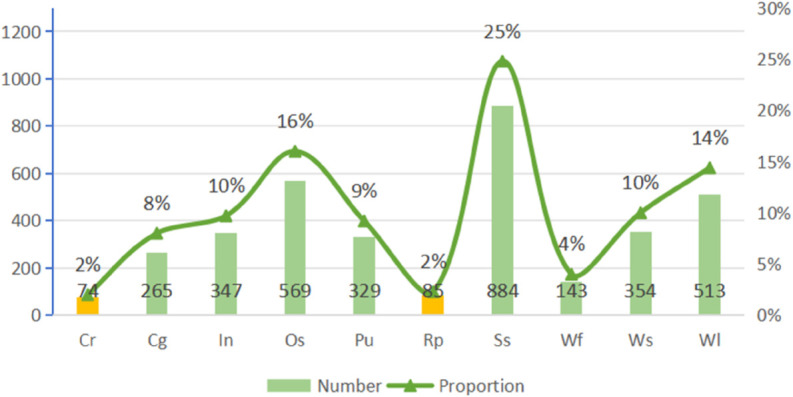
Distribution of categories in the GC10-DET dataset.

The GC10-DET [36] dataset is a publicly available resource specifically curated and annotated to include typical samples of industrial steel surface defects. This dataset encompasses ten distinct types of surface defects: Pu (Punching), Wl (Welding line), Cg (Crescent gap), Ws (Water Spot), Os (Oil spot), Ss (Silk spot), In (Inclusion), Rp (Rolling pit), Cr (Crease), and Wf (Waist folding), as shown in Fig 8. Each defect varies in shape, size, and complexity, thereby comprehensively representing the common steel defects encountered in the industry. Renowned for its high image quality and meticulous annotation, the GC10-DET dataset is frequently employed as a standard benchmark in research related to steel defect detection, as well as in deep learning and computer vision methodologies. The dataset includes defect annotation information with bounding boxes, which facilitates the training and evaluation of models. Considering the variety of defect types and forms available, this dataset offers considerable practical importance for the development and evaluation of algorithms aimed at defect detection, especially in assessing models based on deep learning for object detection. The count of instances for each category is shown in Fig 9.

The Welding Defect Dataset is a high-quality dataset designed to support research and applications in the detection of surface welding defects. It consists of 2,154 images, including 1,619 training images, 409 validation images, and 126 test images. The dataset covers three categories of weld quality: Bad Weld, Good Weld, and Defect. Bad Weld refers to welds that do not meet quality standards, Good Weld indicates welds with acceptable quality, and Defect represents internal or external defects that may occur during the welding process, such as cracks, porosity, or slag inclusions.

### Result

#### Model performance on the NEU-DET dataset.

Due to variations in lighting and differences in materials, the defect images in the NEU-DET dataset exhibit changes in grayscale, resulting in significant visual disparities among intra-class defects, while inter-class defects display similar characteristics. These features present both challenges and opportunities for models, enhancing the precision of validation on the NEU-DET dataset and providing substantial practical value for real-world applications. Throughout the training session, we conducted 250 epochs, during which the trends in loss and precision became evident. Initially, during the first few dozen iterations, the loss values decreased significantly, reaching 0.3673 for the giou_ loss function and 0.3420 for the l1_ loss function. Concurrently, the precision improved markedly, achieving a value of 91.72%. As the iterations progressed, the loss stabilized, and the precision gradually improved until the model converged, as illustrated in Fig 9. Ultimately, the model achieved a precision of 91.72% and a recall of 0.7844, as presented in Table 2.

**Table 2:** The detection performance of different categories on NEU-DET dataset.

Class	precision	Recall	mAP@0.5	mAP@0.5:0.95
Crazing	77.6%	0.239	56.1%	29.5%
Inclusion	70.3%	0.733	72.4%	23.2%
Patches	97.4%	0.93	99.5%	62.1%
Pitted_surface	86.2%	0.875	96.2%	69.9%
Rolled-in_scale	76.3%	0.66	78.6%	35.7%
Scratches	90.7%	0.857	95.3%	45.5%

As illustrated in Table 2, the model demonstrated satisfactory precision across six distinct types of defects, with the Inclusion category recording the lowest precision at 70.3%. In terms of recall, the Patches category achieved the highest score of 0.93, while other categories also exhibited commendable results; however, the Crazing category had the lowest recall at a mere 0.239. This lower recall can be attributed to the lighter color of Crazing, which tends to blend with the background, thereby reducing the recall rate. Due to the feature similarity between Crazing and Scratches, misclassification may occur when one of the categories has insufficient training samples and limited learning capacity. Furthermore, the impact of dataset scale on model performance is significant. According to the mAP metric, the mAP@0.5 precision for Patches, Pitted _ surface, and Scratches exceeded 90%, resulting in an overall mAP@0.5 of 83.03% and an mAP@0.5:0.95 of 45.55%. The high contrast between cracks and the background contributes to their relatively superior precision. As shown in Fig 11, the confusion matrix highlights that the model performs well in category classification. However, it remains sensitive to slight background variations, which underscores both its strength in detail recognition and its susceptibility to subtle noise.

**Fig 10:**
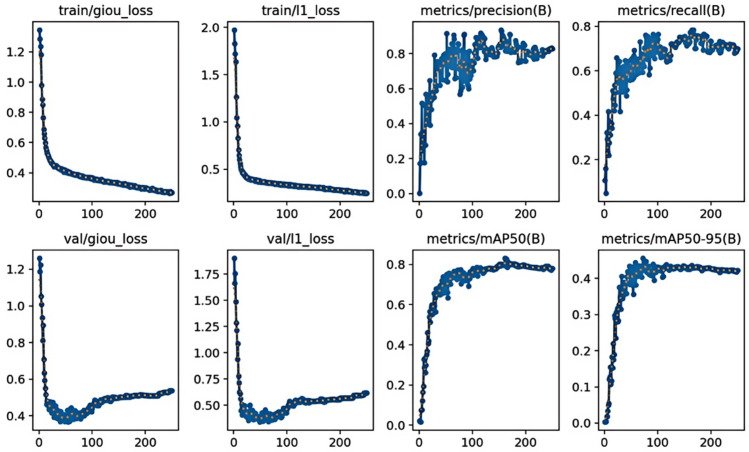
Loss function curve and detection precision experimented on NEU-DET dataset.

**Fig 11:**
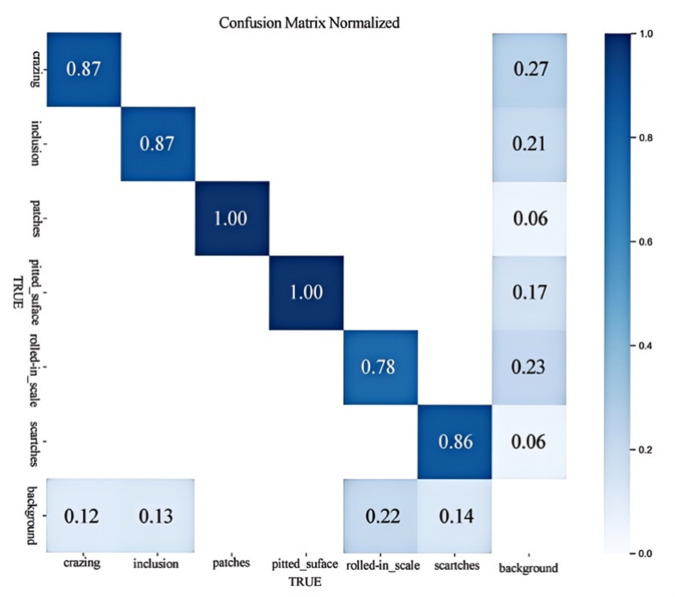
Confusion matrix of the detection results on NEU-DET dataset.

#### Model performance on the GC10-DE dataset and welding defect dataset.

**Fig 12:**
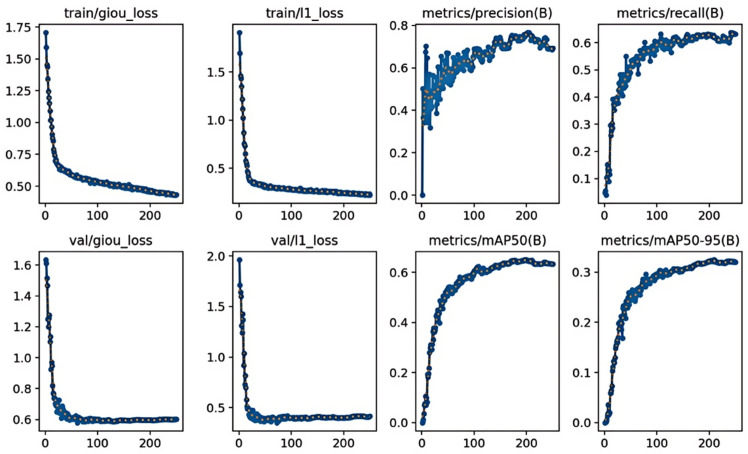
Loss function curve and detection precision experimented on GC10-DE dataset.

To further evaluate the performance of our model, we conducted experiments on the GC10-DET dataset, which comprises a collection of surface defect images sourced from real industrial settings. This dataset contains 3,570 images with a resolution of 2048 x 1000 pixels, partitioned into training and validation sets in an 80:20 ratio. We conducted 250 epochs, and the experimental outcomes are presented in detail. As shown in Table 3, our model demonstrated commendable efficacy on the GC10-DET dataset, achieving a precision of 76.73% and a recall rate of 0.6384. The values for the two loss functions were reduced to 0.5795 and 0.3526, respectively, as illustrated in Table 3, the Loss Function Curve and detection Precision are shown in Fig 12. Although the GC10-DET dataset originates from authentic industrial environments, resulting in potentially lower precision compared to other datasets, it holds greater relevance for actual steel surface defect detection. The Table 4 indicates that various types of defects achieved satisfactory recognition precision within the model, with the ‘Wf’ category attaining an precision as high as 92.2%. In terms of the mAP@0.5 metric, ‘Pu’, ‘Wl’, and ‘Cg’ defects exceeded an precision of 90%, while ‘In’, ‘Rp’, and ‘Cr’ the lowest mAP@0.5 at 28.3%, 24.4%, and 24.7%, respectively. The overall category’s mAP@0.5 reached 65.03%.

**Table 3:** The detection performance for different dataset.

Dataset	Recall/%	Precision/%	mAP@0.5	mAP@0.5:0.95	GIoU Loss	L1 Loss	FPS
NEU-DET	78.44	91.72	83.03%	45.55%	0.3673	0.3420	64.6
GC10-DE	63.84	76.73	65.03%	32.46%	0.5795	0.3526	154
Welding Defect	91.39	92.28	96.04%	84.71%	0.1388	0.0895	120.5

**Table 4:** The detection performance for different categories on GC10-DE dataset.

Class	precision	Recall	mAP@0.5	mAP@0.5:0.95
Pu	91.6%	0.944	94.9%	53.1%
Wl	88.3%	0.965	94.6%	53.7%
Cg	97.9%	0.98	96.3%	61%
Ws	90.8%	0.737	77.5%	45.7%
Os	73.5%	0.627	64.6%	28.2%
Ss	74.4%	0.458	55.7%	23.3%
In	51.9%	0.326	28.3%	9.48%
Rp	72.4%	0.235	24.4%	11.9%
Cr	49.6%	0.2	24.7%	11.3%
Wf	92.2%	0.656	79.3%	36.6%

From the Table 4, it can be observed that due to the limited number of samples for the ‘Cr’ and ‘Rp’ categories in the GC10-DE dataset, their mAP@0.5 values are only 24.7% and 24.4%, respectively, indicating that the model fails to accurately localize true targets in these categories. Although Rp achieves a relatively high precision of 72.4%, its recall is as low as 0.235, resulting in a low mAP@0.5 of 24.4%. This suggests that the model is largely incapable of detecting true Rp instances. These results highlight the model’s insufficient learning of complex defect representations under data-scarce conditions. Furthermore, it reveals a vulnerability to structural similarity interference: Rp defects, characterized by regular indentations, are highly similar in appearance to normal stamping marks, making it difficult for the model to distinguish them based solely on local grayscale features. In comparison, when the number of training samples is sufficient, categories such as Pu and Wl achieve the highest mAP@0.5:0.95 scores, both exceeding 53%, demonstrating the model’s improved discriminative ability under data-rich conditions. In comparison, when the number of training samples is sufficient, ‘Pu’ and ‘Wl’ achieve the highest precision in mAP@0.5:0.95, exceeding 53%, surpassing the average precision of 32.46% (GC10-DE @0.5:0.95). The mAP@0.5:0.95 data indicate that precision is profoundly affected by background context. In real-world scenarios, identifying defects on steel surfaces can be influenced by background noise; thus, enhancing the sample size for particular categories and maintaining a balanced distribution of samples within those categories may enhance the model’s ability to generalize effectively. Among the ten categories, our model successfully identified cracks and their locations, effectively distinguishing them from the background, which underscores the model’s robustness and generalization ability.

To further evaluate the generalization capability and robustness of the proposed model, we conducted validation experiments on the Welding Defect dataset. As shown in Table 3, the model achieved a recall of 91.39% and a precision of 92.28%, indicating that it can effectively capture nearly all real-world welding defects. The detection accuracy, measured by mAP@0.5, reached 96.04%, validating the model’s high precision and robustness in identifying steel surface defects. Moreover, the model attained an mAP@0.5:0.95 of 84.71%, demonstrating strong adaptability to varying defect scales and blurred boundary conditions. The loss function metrics, including GIoU loss and L1 loss, also exhibited favorable values, confirming the model’s efficient learning capability. Table 5 presents a detailed comparison of detection performance across different categories on the Welding Defect dataset. Notably, for the "Good Weld" category, the proposed model achieved an mAP@0.5 of 97.7%, a recall of 93.2%, and a precision of 0.892, further highlighting the strong generalization performance of our approach.

**Table 5:** The detection performance for different categories on welding defect dataset.

Class	Precision	Recall	mAP@0.5	mAP@0.5:0.95
Bad Weld	88.3%	87.5%	95.1%	85.9%
Good Weld	89.2%	93.2%	97.7%	87.8%
Defect	88.2%	89.9%	95.0%	80.4%

**Fig 13:**
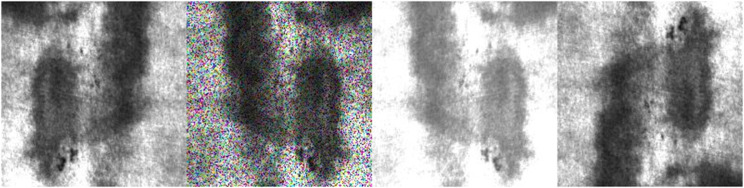
Examples of images processed with different data augmentation methods.

**Fig 14:**
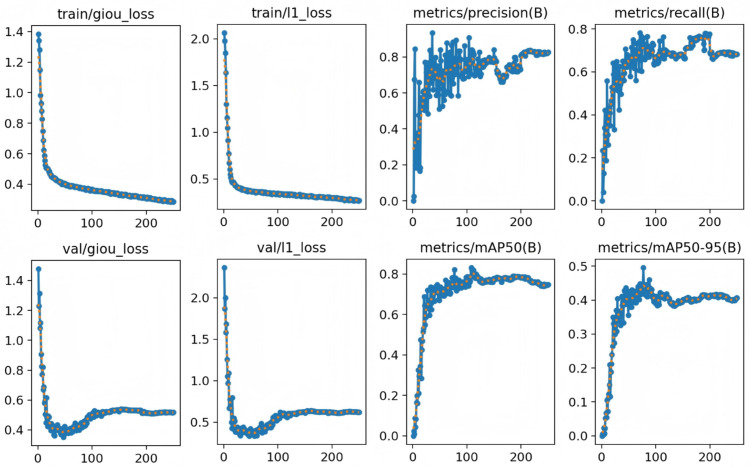
Loss curves and detection precision for processed images on the GC10-DE dataset.

To verify the potential of the proposed model for future applications in real-world industrial scenarios with data imbalance, we performed data augmentation on the NEU-DET dataset. Specifically, we applied brightness enhancement, rotation, and noise interference, which expanded the dataset size by three times, as shown in Fig 13. From the training results after augmentation, the model demonstrated strong learning ability and generalization. The training loss decreased rapidly at the early stage and then stabilized. The validation loss converged simultaneously and closely matched the training curve. This indicates that the augmentation effectively improved the model’s generalization ability without causing overfitting. As shown in Fig 14, the key evaluation metrics, precision and recall, both reached and maintained high levels. This suggests that the model achieved excellent detection performance on clear targets. However, the mAP50-95 remained around 0.45. The mAP50 showed a slight decrease compared to the previous results. The reason is that random brightness changes and intensive noise interference during augmentation affected some targets, making them harder to recognize completely. In summary, data augmentation successfully enriched the dataset features and promoted robust learning of the model. Nevertheless, further optimization of augmentation strategies is needed to better handle challenging samples.

**Fig 15:**
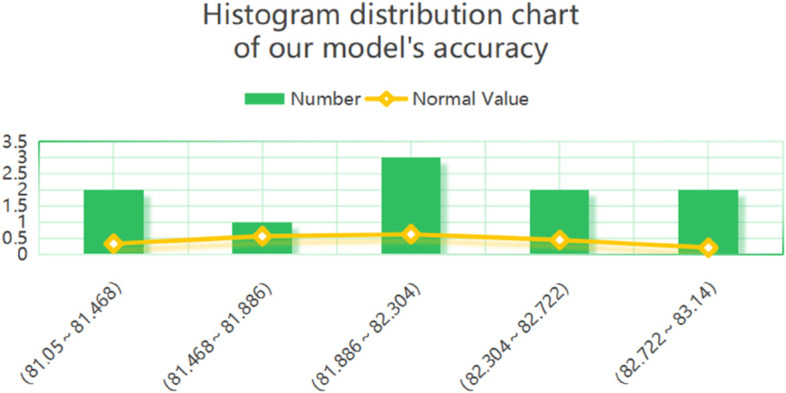
Histogram of model accuracy training results.

Because the experimental results may be affected by randomness, we conducted repeated experiments and constructed a normal histogram, as shown in Fig 15. The figure shows that the data overall exhibit a pronounced conformity to a normal distribution. Detection accuracy is concentrated in the range of 81.886 to 82.304, with a frequency of three occurrences, indicating that the model outputs are most stable within this interval. The normal curve is smooth and symmetrically decreases on both sides of this central range, closely matching the distribution of the histogram, which further confirms the normality of the data. The frequencies in the side intervals, such as 81.05–81.468 and 82.722–83.14, are relatively low, suggesting that outliers or discrete points are rare and that the model outputs are consistent. This distribution indicates that the model possesses high predictive robustness and reliability. If applied to quality control scenarios, it can be considered that the current process is under control, with no need for substantial adjustments.

#### Comparison experiment.

In this research, a range of comparative experiments were performed using the public NEU-DET dataset, with results presented in the table below. The proposed approach was evaluated against various leading models, such as ScaledYOLOv4-csp [37], YOLO-MSFE-EFF [38], Yuan’s model [39], YOLOv7-tiny [40], Mask-R-CNN [41,42], and YOLOv7 [43], as detailed in Table 6. The findings reveal the superior efficacy of our approach in both the mAP@0.5 and mAP@0.5:0.95 metrics. Data in the table indicates that the parameter count of our model is slightly higher than that of YOLOv7-tiny and YOLO-MSFE-EFF. Remarkably, our model attained an mAP@0.5 of 83.30% and an mAP@0.5:0.95 of 45.55%, exceeding the established YOLOv7 model by margins of 11.06% and 8.35%, respectively, on the NEU-DET dataset. With a total of just 15.78M parameters, our model is more lightweight in comparison to other foundational state-of-the-art models.

**Table 6:** Comparison of our proposed model with other state-of-the-art models on NEU-DET dataset.

Methods	Params (M)	FLOPs (G)	mAP@0.5	mAP@0.5:0.95
ScaledYOLOv4 - csp [37]	52.53	119.4	69.37%	33.57%
YOLOv7 [43]	37.22	104.8	71.92%	37.2%
YOLO - MSFE - EFF [38]	9.54	18	73.08%	37.57%
Yuan’s model [39]	18.23	——	78.2%	22.46%
YOLOv7 - tiny [40]	6	——	73.9%	——
Mask - R - CNN [41,42]	73.6	146.9	81.2%	35.3%
Fast RCNN [10]	100.02	134.4	76.7%	32.6%
MobileViTv2 - YOLOv8 [32]	27.5	34.9	74.1%	——
DETR	36.7	——	66.6%	—— %
LRT - DETR	6.78	13.7	74.8%	——
ours	16.66	45	83.03%	45.55%

Regarding mAP@0.5 and mAP@0.5:0.95, our model outstrips the Mask-R-CNN model by 1.83% and 10.25%, respectively, while also having 56.94M fewer parameters than Mask-R-CNN. To validate the effectiveness of our proposed model, we conducted a comprehensive comparative analysis within the RCNN framework. Specifically, in comparison to Fast RCNN, our model not only significantly reduces the number of parameters but also achieves higher accuracy. Remarkably, while drastically decreasing the parameter scale, our model surpasses Fast RCNN by 6.33% in the mAP@0.5 metric, thereby demonstrating its superiority and efficiency. The parameter counts for the models YOLOv7-tiny, YOLO-MSFE-EFF, and Yuan’s increase in succession, and their mAP performance improves in tandem. Our solution not only exceeds traditional YOLO models but also demonstrates greater efficiency concerning parameter count. Our model demonstrates superior performance across various architectures, including the YOLO series, different variants of R-CNN, and multiple Transformer-based models. Specifically, it performs exceptionally well with MobileViT v2-YOLOv8 and variants of DETR. Notably, based on the original DETR model, our approach achieves significant improvements in recognition accuracy while drastically reducing the number of parameters. We also introduce a novel combination of channel shuffle and the SimAM attention mechanism. This helps improve interconnections between encoding units and enhances key feature representations in Transformer models.

In the LRT-DETR model, the substantial reduction in parameters and computational load presents challenges for executing high-precision tasks, resulting in an accuracy that is 8.23% lower than that of our proposed model.

To further demonstrate the superiority of our proposed model, we conducted a comprehensive comparison with state-of-the-art detection methods on each defect category of the NEU-DET dataset. The comparison results are presented in Table 7. Our improved model achieved outstanding performance in both per-class Average Precision (AP) and mean Average Precision at IoU threshold 0.5 (mAP@0.5), reaching an mAP@0.5 of 83.03%, which is 1.8% higher than the second-best PMSA-DETR.In terms of per-class detection accuracy, our model achieved the highest AP for the Pa and Rs categories among all compared methods, with scores of 97.4% and 76.3%, respectively, highlighting the effectiveness of our model in collaborative category optimization. Among the YOLO series, YOLOv9 performed best, with an mAP@0.5 of 79.2%, still 3.8% lower than our model. For individual categories, YOLO11 achieved the highest AP in the Cr category, yet its overall performance remained inferior to ours. Within the DETR-based models, PMSA-DETR achieved the highest mAP@0.5 at 81.2%, which is 1.8% lower than our model. The traditional detection model Faster R-CNN yielded a relatively low mAP@0.5 of 74.8%, with inferior performance in both per-class accuracy and overall detection precision.In summary, these results demonstrate that our model exhibits superior accuracy and robustness in the complex task of steel surface defect detection.

**Table 7:** Comparison of detection methods by defect category on NEU-DET dataset.

Methods	Cr	In	Pa	PS	RS	Sc	mAP@0.5
YOLOv5 [44]	65.9	67.2	91.1	87.1	56.3	93.0	75.1
YOLOv8 [45]	70.7	75.6	90.8	89.3	69.7	94.6	78.4
YOLOv9 [46]	74.2	70.0	91.1	90.5	66.1	92.3	79.2
YOLOv10 [47]	75.3	69.0	91.4	86.7	71.7	90.2	78.1
YOLO11 [48]	77.5	69.8	93.8	91.8	69.5	94.9	78.7
Faster RCNN	72.4	69.9	91.9	81.8	59.8	93.2	74.8
REDef-DETR [49]	75.5	67.7	92.6	99.5	75.2	88.2	80.6
SAM-DETR [50]	71.6	63.7	84.9	69.1	60.4	88.0	71.3
DAB-DETR [51]	69.3	66.8	88.8	73.8	61.5	90.8	73.5
DN-DETR [52]	74.1	64.5	86.9	71.0	59.3	87.4	72.2
PMSA-DETR [53]	74.9	68.6	92.9	84.5	70.5	95.7	81.2
Ours	77.6	70.3	97.4	86.2	76.3	90.7	83.0

Following the comparisons made with the NEU-DET dataset, we then carried out additional assessments using the GC10-DET dataset. We compared with the start of the art models for each target detection category, the comparison results are shown in Table 8. Based on the evaluation conducted on this dataset, our proposed model achieved a mAP@0.5 of 65.0%, which is comparable to the best-performing model among the baselines, PMSA-DETR. In addition, the proposed model outperformed comparative methods in eight detection categories (Cg, Ss, Os, Wl, Ws, Wf, In), particularly for categories with generally lower detection accuracy. Notably, the detection precision for Os and In reached 73.5% and 51.9%, respectively, representing the highest values among all compared models. The YOLO series exhibited noticeable variability in detection performance. While YOLOv8, YOLOv9, and YOLOv11 achieved mAP@0.5 scores in the range of 63.3% to 64.4%, YOLOv10 yielded a substantially lower score of 60.0%, indicating reduced suitability for small object detection tasks in this context. Among the DETR-based models, PMSA-DETR and DN-DETR achieved the highest mAP@0.5 values of 65.0% and 64.9%, respectively. However, in specific categories such as Ss and Os, models like DAB-DETR and DN-DETR showed up to 10% lower detection accuracy compared to our model. The conventional Faster R-CNN model demonstrated poor performance in categories such as Rp and Wf, and its overall mAP was also comparatively lower. The proposed model improves fine-grained detection accuracy through architectural optimization, and experimental results demonstrate its robustness and effectiveness in handling complex defect detection scenarios.

**Table 8:** Comparison of detection methods by defect category on GC10-DET dataset.

Method	Cg	Ss	Os	Wl	Pu	Ws	Rp	Wf	Cr	In	mAP@0.5
YOLOv5	82.1	78.2	65.4	81.4	78.2	83.6	68.4	75.1	37.8	26.6	62.5
YOLOv8	97.1	55.8	66.0	88.4	93.6	74.4	66.8	93.3	45.4	31.6	63.3
YOLOv9	96.9	68.1	62.1	87.5	88.0	84.3	70.1	84.9	51.3	30.7	64.4
YOLOv10	93.3	53.7	65.5	72.8	91.8	66.9	68.7	85.1	37.4	34.9	60.0
YOLOv11	97.4	54.1	66.9	88.8	95.8	70.1	65.2	91.1	47.8	34.5	64.2
Faster R-CNN	89.1	62.4	55.8	78.7	72.8	72.7	66.2	66.9	37.7	32.6	58.9
SAM-DETR	95.6	56.2	59.9	85.0	92.5	87.1	77.3	83.5	50.8	48.9	64.5
DAB-DETR	97.2	60.8	69.3	91.9	91.8	88.6	71.6	86.4	48.9	50.5	64.8
DN-DETR	97.5	56.8	70.8	82.8	90.8	90.5	67.7	87.0	47.5	49.6	64.9
PMSA-DETR	97.8	69.7	68.9	86.2	90.7	87.2	72.9	90.2	48.8	34.8	65.0
Ours	97.9	74.4	73.5	88.3	91.6	90.8	72.4	92.2	49.6	51.9	65.0

#### Ablation and visualization experiments.

In this section, we present ablation studies aimed at determining the contribution of each module within our model. The results of these experiments are summarized in Table 9. Model A serves as the baseline, integrating a ResNet18 backbone network with a Transformer, achieving mAP@0.5 and mAP@0.5:0.95 of 79.40% and 43.55%, respectively. Building upon Model A, Model B incorporates the SH-encoder module proposed in this study, which utilizes single-channel convolution and channel shuffling. This approach not only reduces the parameter count by 4 million but also enhances inter-channel interaction, resulting in increases of 2.24% and 0.67% in mAP@0.5 and mAP@0.5:0.95, respectively, compared to the baseline model, while significantly decreasing the computational load and FPS remains stable.

**Table 9:** Impact of each module on the model in ablation studies.

Model	Model structure	Params (M)	FLOPs (G)	mAP@0.5	mAP@0.5:0.95	FPS
A	Resnet18 + transformer	20	58.6	79.40%	43.55%	68.9
B	A + SH - encode	16	44.6	81.64%	44.22%	67.5
C	B + UPC - SimAM(ours)	16	45	83.03%	45.55%	64.6
D	C + an additional encoder	17	45.7	77.34%	43.80%	42.7
E	Resnet50 + transformer	42	136	77.79%	44.78%	40.7

Model C embodies our proposed all-encompassing framework that further integrates feature fusion across multiple dimensions and adopts parameter-free attention modules built on the principles of Model B. By merging features from various scales and utilizing a mechanism for attention that does not require parameters, we successfully managed to limit the expansion of model parameters while simultaneously improving the interaction among features. This strategy yielded enhancements in mAP@0.5 and mAP@0.5:0.95, reaching values of 83.03% and 45.55%, respectively. From the parameters, FLOPs, and FPS, it can be seen that the computational amount and complexity of the model remain almost unchanged. The data demonstrates that omitting these essential modules results in different levels of performance decline, highlighting their importance for the model’s overall performance.

In our experiments involving the CNN-based module E, we selected ResNet50 as the convolutional embedding backbone network. The results indicate that, despite a significant increase in both parameter count and computational load on the NEU-DET dataset, the improvement in precision was not substantial. Specifically, the mean Average Precision (mAP) at IoU thresholds of 0.5 to 0.95 was only 1.23% higher than that of the benchmark model A. The excessive parameter count and computational demands may limit the model’s practical applicability. The FPS dropped significantly to 40.7. Consequently, we chose ResNet18 as our backbone network, as it offers a more favorable balance between parameter efficiency and precision.

Expanding on Model C, we developed Model D to examine if augmenting the number of encoders would improve the precision of the model. Our experiments reveal that repeatedly stacking the Transformer block leads to suboptimal outcomes. In particular, there is a 5.69% drop in mAP@0.5, and the mAP@0.5:0.95 diminishes by 1.75%. Due to the substantial increase in computational load introduced by the encoder, the model’s FPS is 21.9 lower compared to ours. These results reinforce the efficacy of our model’s architecture and provide important guidance for forthcoming enhancements.

**Fig 16:**
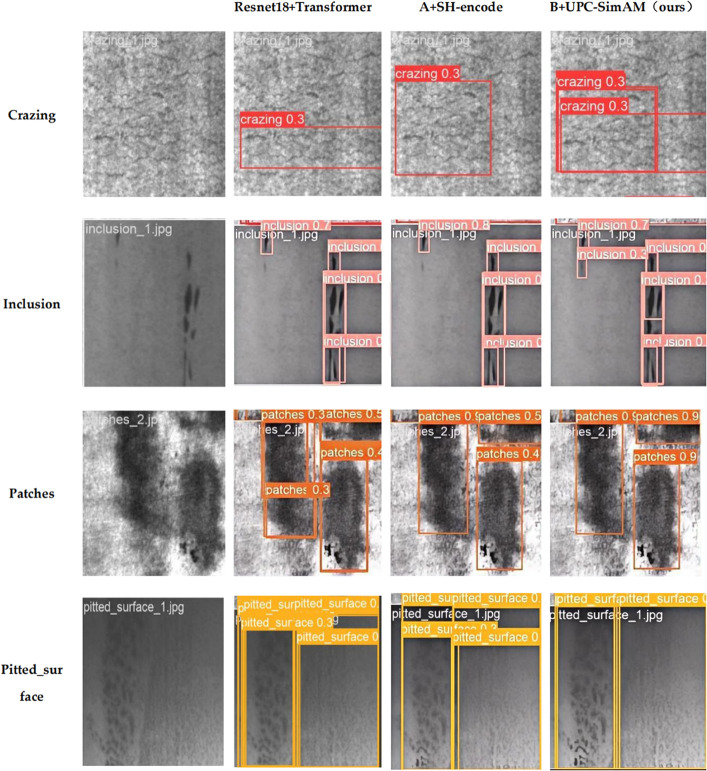
Visualization results of different modules in ablation studies.

Following the precision analysis of individual modules, we conducted a comparative analysis through the visualization of detection bounding boxes and categories on the NEU-DET dataset, as illustrated in Fig 16. The visualizations demonstrate that our model not only accurately identifies defect categories, such as cracks, but also achieves a high level of recognition precision. Taking ‘crazing’ as an example, when compared to the original image, the baseline model was able to recognize the defect category; however, the incorporation of the SH-encoder module expanded the detection scope. Within our proposed comprehensive model, the integration of the UPC-SimAM module further broadened the detection range, indicating an enhancement in the model’s capacity to manage similarly interfering backgrounds. In the ‘inclusion’ category, the detection of cracks showed significant improvement with the addition of various modules, enabling the model to identify minor cracks that had previously evaded detection. This enhancement considerably boosts the model’s ability to recognize smaller targets, thereby validating its precision. Regarding the ‘patches’ category, the baseline model exhibited redundancy and repetition in target identification, accompanied by a suboptimal range of identified categories. In the observation of pitted-surface cracks, our proposed module demonstrates significant effectiveness. It not only maintains a high level of recognition accuracy but also greatly reduces the number of redundant detection boxes. Conversely, the model we propose delineates the location and dimensions of cracks with greater accuracy, highlighting the strong performance of our modules in feature extraction and processing abilities.

In fine mesh-like images, all evaluated models were only able to localize partial fragmented regions but failed to accurately detect the entire crack structure. This limitation is attributed to the high similarity between crack features and the background texture, making it challenging for models to distinguish cracks from the metal substrate patterns. Meanwhile, although other crack detection methods achieved relatively high precision, Fig 16 reveals potential risks of missed detections and duplicate detections. Such deviations are mainly caused by the pre-improvement models’ overreliance on local grayscale transition features, which hindered adequate learning of the feature differences between inclusions and indentations. Our proposed model addresses these issues by introducing novel modules that enhance the structural feature disentanglement capability and multi-scale semantic fusion ability.

To summarize, the visual outcomes not only demonstrate the model’s ability to recognize various defect categories but also indicate that incorporating the SH-encoder and UPC-SimAM modules gives our model a distinct edge in handling intricate backgrounds and subtle defects. This improvement greatly enhances the model’s overall performance and accuracy.

## Discussion and conclusion

In this research, we present a novel method rooted in deep learning aimed at detecting surface imperfections in steel. This approach is specifically designed to address the complexities tied to these imperfections and the challenges involved in feature extraction across various scales. To address these obstacles, we developed a multi-scale feature extraction module that utilizes both Transformers and CNNs, employing convolutional kernels of varying sizes to efficiently capture features at different scales. Furthermore, we have developed a streamlined channel-mixing encoder component aimed at reducing the problem of feature loss and boosting the interplay between different features. The integration of the UPC-SimAM module has strengthened the model’s feature fusion capabilities, while SimAM improves CNN performance through a capability weighting strategy. We also refined the backbone network to boost the model’s efficiency in feature extraction. Through ablation studies conducted on the publicly available NEU-DET and GC10-DE datasets, we validated the efficacy of our model. Furthermore, comparisons with various leading object detection frameworks highlighted the benefits of our approach. The outcomes of our experiments demonstrated our model’s outstanding performance, especially regarding the mAP@0.5 and mAP@0.5:0.95 metrics.

While the proposed model has shown notable outcomes in detection, the extensive number of parameters inherent in Transformers creates challenges for real-time detection when applied to computational devices in actual industrial environments. Therefore, focusing on lightweight Transformer models or employing techniques like distillation, pruning, and quantization to enhance the detection speed of the model is crucial for future research [54]. Given the significant progress made in CNNs, we are optimistic that, with adequate development, Transformers will find a practical balance between the parameter count and detection speed. In our upcoming initiatives, we plan to optimize the model’s architecture and enhance its ability to detect defects, particularly on steel surfaces. For rare defect categories with limited samples, such as the ‘Cr’ class in the GC10-DET dataset, future work will focus on few-shot learning and synthetic data augmentation strategies. Specifically, we plan to employ cross-domain meta-learning frameworks to rapidly adapt to a small number of real samples, as well as generate realistic defect data based on physical deformation principles, aiming to overcome the bottleneck of low detection accuracy caused by scarce training data. We will explore methods for model compression and acceleration to make the model more suitable for real-world industrial usage, while maintaining high levels of accuracy and efficiency. Through these endeavors, we aim to provide more efficient and applicable solutions for defect detection on steel surfaces
